# Running rich: how excess fatty acid oxidation drains the cardiac engine

**DOI:** 10.1172/JCI204459

**Published:** 2026-05-01

**Authors:** Steven M. Claypool, Carla M. Koehler

**Affiliations:** 1 Department of Physiology, Pharmacology and Therapeutics, Johns Hopkins University School of Medicine, Baltimore, Maryland, USA.; 2 Department of Chemistry and Biochemistry, Molecular Biology Institute, and Jonsson Comprehensive Cancer Center, UCLA, Los Angeles, California, USA.

## Abstract

Fatty acid oxidation (FAO) provides the healthy heart with 60%–90% of its ATP, with the remainder coming from metabolism of glucose. Metabolic flexibility is key to heart function, ensuring an uninterrupted source of fuel. In heart failure, a shift from FAO to glucose-dependent metabolism occurs as disease progresses, supporting the widely held notion that fat is the optimal substrate in the heart. In this issue of the *JCI*, Kim et al. challenge this assumption. In studies of acetyl-CoA carboxylase–deficient (ACC-deficient) mice, they found that unregulated use of fat as a substrate led to cardiac damage. ACC-deficient mice developed cardiolipin deficiency as a result of excessive FAO depleting stores of linoleic acid, which is used as a substrate for cardiolipin maturation. The resulting mitochondrial dysfunction was associated with dilated cardiomyopathy and heart failure in these mice. The findings highlight potential for development of therapeutic strategies that balance energy sources and replenish cardiolipin levels.

## Introduction

The heart, like the brain, is an energy-consuming organ that never stops working. The heart runs primarily on fatty acid oxidation (FAO), which supplies over 70% of its energy (1). For decades, researchers viewed this FAO dependence as the heart’s optimal state, leveraging an evolutionary adaptation that maximizes energy extraction from fat. However, a new study by Kim et al. (2) using mice challenges this assumption, showing that excessive FAO can paradoxically transform the heart’s main fuel into something harmful.

## Cardiac metabolism and the heart failure puzzle

The healthy heart generates over 95% of its ATP via oxidative phosphorylation, with 60%–90% derived from FAO and 10%–40% from glucose (1). This reflects remarkable metabolic flexibility — the capacity to shift among fuel sources in response to workload and availability. This flexibility operates through the Randle cycle (or glucose–fatty acid cycle), where fatty acid and glucose oxidation work in opposition in the context of cardiac metabolism (3). When FAO increases, its by-products block glucose use. Conversely, increased glucose oxidation produces malonyl-CoA, which inhibits carnitine palmitoyltransferase 1 (CPT1), the carnitine shuttle enzyme critical for moving long-chain fatty acids into mitochondria, thus reducing FAO. Acetyl-CoA carboxylase (ACC), the rate-limiting step in FAO, plays a key regulatory role by converting acetyl-CoA to malonyl-CoA (1). Cells have 2 ACC variants: ACC1 in the cytosol contributes to fatty acid synthesis, whereas ACC2 associates with the mitochondrial membrane and controls CPT1. Heart cells have low ACC1 but exceptionally high ACC2, reflecting the need for precise control of FAO. In heart failure, metabolism increasingly favors glucose as disease advances. This adaptation may lower oxygen demand, since fat oxidation requires 10%–15% more oxygen than glucose for the same ATP output.

**Figure 1:**
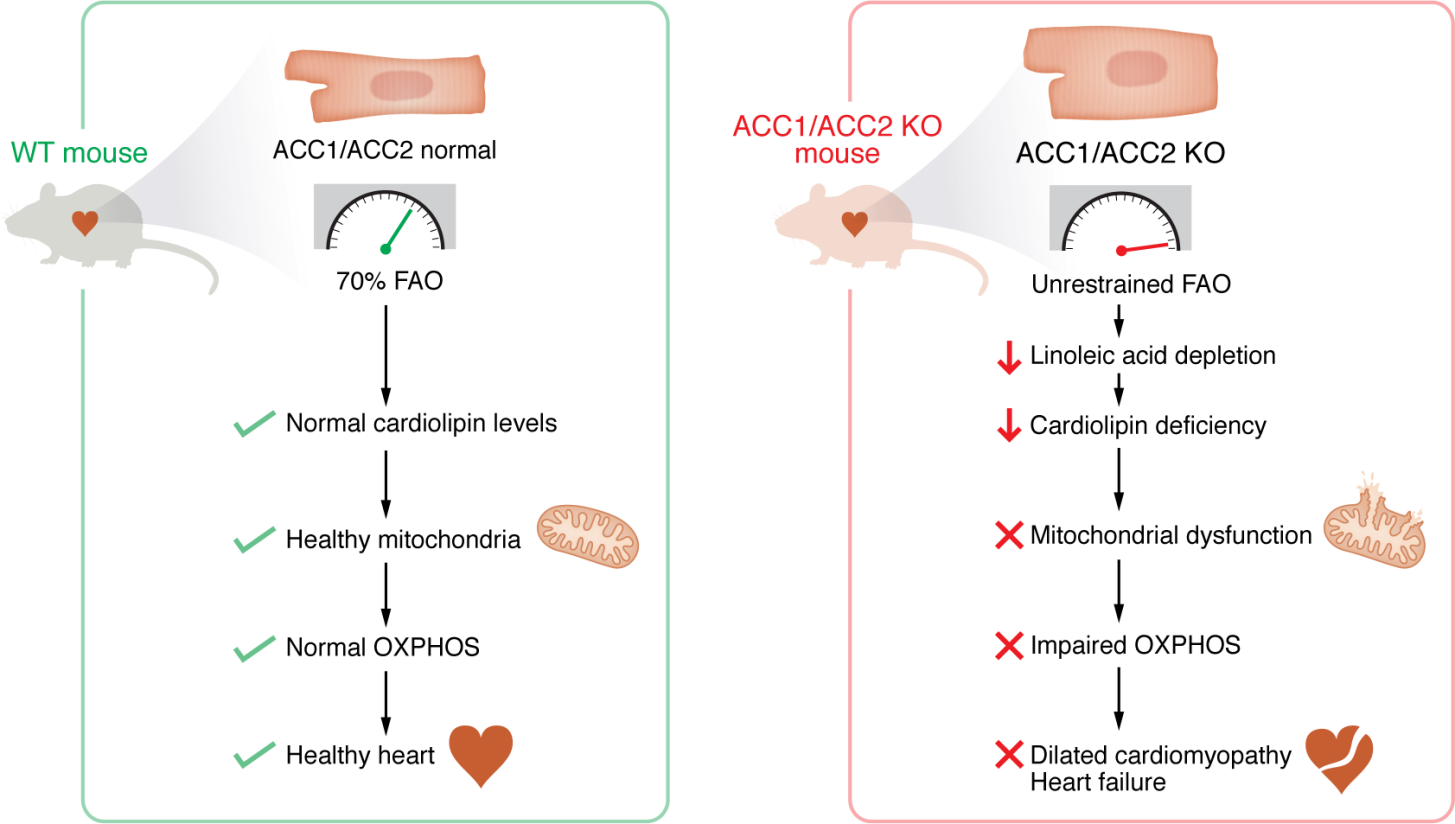
Unrestrained FAO leads to cardiolipin deficiency, mitochondrial dysfunction, and heart failure in mice. Kim et al. generated cardiac-specific ACC1/ACC2 deletion to elicit unrestrained FAO in mice and observed that excessive fat metabolism led to depletion of linoleic acid stores, resulting in cardiolipin insufficiency and, ultimately, mitochondrial dysfunction (2). Mitochondrial dysfunction was associated with dilated cardiomyopathy and heart failure in the ACC1/ACC2 model. These findings challenge widely held assumptions that fat is the optimal substrate in the heart. Rather, they support exploration of therapeutic strategies that balance energy sources and reverse cardiolipin depletion. OXPHOS, oxidative phosphorylation.

One intriguing pattern in cardiovascular medicine is the abovementioned shift in metabolism during heart failure. As hearts weaken, they gradually shift away from FAO and rely more on glucose, a process referred to as the “fetal metabolic shift,” which includes the renewed expression of fetal isoforms of metabolic enzymes that support adverse metabolic remodeling in the postnatal heart (4). This has sparked debate over whether the shift to glucose-based metabolism is harmful or beneficial. The prevailing view favors protection, motivating therapies to reduce FAO and boost efficiency (5). But what happens when FAO regulation breaks down completely? In the present issue of the *JCI*, the study by Kim et al. (2) answers that question by generating mice lacking both ACC1 and ACC2 in heart cells. Removing this metabolic brake forced hearts to burn fat at maximum capacity without modulation. The results were striking (Figure 1): ACC cardiac-specific double-knockout mice developed severe dilated cardiomyopathy by 2 months, with progressive decline into advanced heart failure. Ejection fraction dropped over 50%, hearts enlarged dramatically, and extensive scarring appeared. Mice lacking only 1 ACC variant exhibited milder, delayed heart dysfunction, confirming that the severity of FAO dysregulation directly correlates with cardiac damage.

## The cardiolipin connection and therapeutic rescue

Through comprehensive lipid analysis, the authors traced a direct path from excessive FAO to heart failure. The problem they identified was a surprisingly strong 50% reduction in cardiolipin in ACC-deficient mice, including an 80% decrease in its mature form, tetralinoleoyl cardiolipin, which is prevalent in the heart and essential for mitochondrial function therein. Cardiolipin, primarily residing in the inner mitochondrial membrane, is required for optimal electron transport chain performance (6). Its mature form, produced by a ubiquitous remodeling process that nonetheless establishes distinct tissue-specific cardiolipin acyl chain compositions (7), stabilizes respiratory complexes and enables efficient electron transfer (8). When cardiolipin is deficient, as in Barth syndrome (an inherited mitochondrial disease caused by mutations in the cardiolipin-remodeling enzyme tafazzin), the resulting respiratory complex impairment leads to dilated cardiomyopathy (8). Kim et al.’s study showed that excessive FAO driven by ACC loss created a comparable cardiolipin deficiency through a different route: rather than disrupting cardiolipin remodeling, unchecked FAO depleted the substrate pool, particularly linoleic acid (C18:2)–containing phospholipids that serve as building blocks for cardiolipin maturation (7). This caused dysfunction of the mitochondrial respiratory complexes involved in oxidative phosphorylation, thereby mimicking Barth syndrome through metabolic rather than genetic mechanisms. Isotopic labeling studies linked dysfunction of mitochondrial respiratory complexes to depletion of linoleic acid–containing phospholipids that tafazzin uses as substrates to remodel cardiolipin, not impaired synthesis of nascent cardiolipin. Crucially, linoleic acid uptake was normal, indicating that excessive oxidative consumption was the root cause.

The rescue experiments in this study are particularly compelling. Treatment with etomoxir, which blocks FAO by targeting CPT1 and other proteins involved in fatty acid metabolism and transport (9), completely prevented heart failure, but only when started early. This intervention restored linoleic acid availability, replenished cardiolipin, corrected mitochondrial function, and preserved cardiac performance. However, timing proved critical: early intervention (4 weeks before dysfunction) offered protection, but starting after dysfunction appeared (at 20 weeks) failed to help. This suggests that while preventing FAO-driven damage is achievable, reversing established cardiolipin depletion may require different strategies. The failure of PPARα agonist treatment (10), which increased expression of FAO genes but worsened function, underscores that normalizing gene expression without addressing metabolic imbalance falls short. Notably, dietary linoleic acid supplementation also failed to rescue the phenotype despite increasing cardiac linoleic acid. This shows that simply providing more substrate cannot overcome excessive oxidative consumption.

## Rethinking fat metabolism in heart disease

These findings carry interesting implications for heart failure treatment. Whereas modest FAO inhibition may help in certain conditions, this study warns against strategies that might inadvertently enhance FAO in failing hearts. It reveals an optimal window; too little FAO compromises energy generation, whereas too much FAO disrupts lipid balance and mitochondrial integrity. The observation that failing hearts eventually suppress FAO as disease progresses suggests that the metabolic shift might actually represent an adaptive attempt to limit further damage (4). This reframes the fetal metabolic shift not as pathological regression but as a protective restraint of a potentially destructive process. Kim et al.’s insights extend beyond this knockout model. The work establishes a framework for thinking about the function of metabolic substrates as not just for ATP generation but also for preserving structural lipids essential for mitochondrial function. Hearts must balance burning fatty acids for energy against retaining enough for membrane synthesis and remodeling. This may be particularly relevant in obesity, diabetes, and metabolic syndrome — conditions with chronic lipid overload and potentially elevated cardiac FAO that could, over years, contribute to progressive cardiolipin depletion. Furthermore, this study highlights cardiolipin as a potential biomarker and therapeutic target (11). Current clinical trials examining cardiac metabolism focus heavily on substrate utilization but largely overlook membrane lipid composition. Methods for assessing cardiolipin profiles could provide valuable diagnostic information (12).

This model may be useful for future studies to address fundamental questions about tissue-specific lipid metabolism. Understanding why linoleic acid is uniquely vulnerable to depletion during excessive FAO could reveal important principles about how cells prioritize different fatty acids for energy versus structural functions. Investigating how dietary fats are partitioned between immediate oxidation and membrane incorporation might uncover regulatory checkpoints that could be therapeutically targeted to preserve essential lipids during metabolic stress. Mechanistic studies in fatty acid metabolism and trafficking may yield strategies to maintain optimal mitochondrial function in the heart during aging, disease, and metabolic dysfunction.

Kim et al. offer compelling evidence that the heart’s relationship with FAO is more nuanced than previously thought (13). Rather than strategies to maximize FAO to boost fuel availability, FAO needs tight regulation. Too little FAO compromises energy production; too much depletes cardiolipin and drives heart failure. Future therapeutic strategies should not aim to maximize or minimize FAO but to optimize it, maintaining sufficient activity for energy while preventing excessive oxidation that disrupts lipid balance (5). The complete rescue achieved by early inhibition of FAO demonstrates that this balance can be therapeutically manipulated and underscores both the importance of timing and the challenge of reversing established damage. Of course, careful studies in humans will be needed to translate these insights into the clinic.

## Conflict of interest

The authors have declared that no conflict of interest exists.

## Funding support

This work is the result of NIH funding, in whole or in part, and is subject to the NIH Public Access Policy. Through acceptance of this federal funding, the NIH has been given a right to make the work publicly available in PubMed Central.

- NIH grants R01HL165729 and R01GM151746 to SMC and R01GM061721 and R01GM037981 to CMK.

## Version 1 05/01/2026

Electronic publication
